# Supplementary material for: Seed survival of Australian Acacia in the Western Cape of South Africa in the presence of biological control agents and given environmental variation
Source: PeerJ. 2019 Apr 29;7:e6816. doi: 10.7717/peerj.6816 (PMC6497107; doi:10.7717/peerj.6816)
Supplement: Supplemental Information 1 [file peerj-07-6816-s001.docx]

## Supplementary material

### **Proportion of seeds initially assessed as surviving and aborted**

The proportion of seeds initially assessed as aborted and surviving without insect damage for each species was calculated as follows:

$$AI=\frac{ASB-ASI}{ASB}$$

where *AI* is the proportion of aborted seeds without insect damage, *ASB* is the aborted seeds subsample and *ASI* is the aborted seeds with insect damage in the subsample;

$$SI=\frac{SSB-(SSI\times NV)}{SSB}$$

where *SI* is the proportion of surviving seeds without insect damage, *SSB* is the surviving seeds subsample, *SSI* is the surviving seeds with insect damage in the subsample and *NV* is the proportion of non-viable surviving seeds with insect damage.

### **Proportion of seeds aborted, surviving and predated per tree**

1. *Proportion of seeds aborted per tree:*

$$PA=\frac{SA \times AI}{TS}$$

where *PA* is the estimated proportion of seeds aborted per tree, *SA* is the sum of seeds initially assessed as aborted, *AI* is the proportion of seeds assessed as aborted without insect damage and *TS* is the total seeds assessed (aborted + predated + surviving);

1. *Proportion of seeds surviving per tree:*

$$PS=\frac{SS \times SI}{TS}$$

where *PS* is the proportion of seeds surviving per tree, *SS* is the sum of seeds initially assessed as surviving, *SI* is the proportion of seeds assessed as surviving without insect damage and *TS* is the total seeds assessed;

1. *Proportion seeds predated per tree:*

$$PP=\frac{SP+\left( SS\times SWI \right)+\left( SA\times AWI \right)}{TS}$$

where *PP* is the proportion of seeds predated per tree, *SP* is the sum of the seeds initially assessed as predated, *SS* is the sum of seeds initially assessed as surviving, *SWI* is the proportion of seeds assessed as surviving with insect damage (1 - *SI*), *SA* is the sum of seeds initially assessed as aborted, *AWI* is the proportion of seeds assessed as aborted with insect damage
(1 - *AI*) and *TS* is the total seeds assessed.

### Total aborted, predated and surviving seeds per tree

$$A= \left( p \times s \right) \times PA$$

where *A* is the total seeds aborted per tree, *p* is the estimated pods per tree, *s* is the average seeds per pod and *PA* is the proportion of aborted seeds per tree;

$$P= \left( p \times s \right) \times PP$$

where *P* is the total seeds predated per tree, *p* is the estimated pods per tree, *s* is the average seeds per pod and *PP* is the proportion of predated seeds per tree;

$$S= \left( p \times s \right) \times PS$$

where *S* is the total seeds surviving per tree, *p* is the estimated pods per tree, *s* is the average seeds per pod and *PS* is the proportion of surviving seeds per tree.
