# Supplementary figures and images for: Seed survival of Australian Acacia in the Western Cape of South Africa in the presence of biological control agents and given environmental variation
Source: PeerJ. 2019 Apr 29;7:e6816. doi: 10.7717/peerj.6816 (PMC6497107; doi:10.7717/peerj.6816)

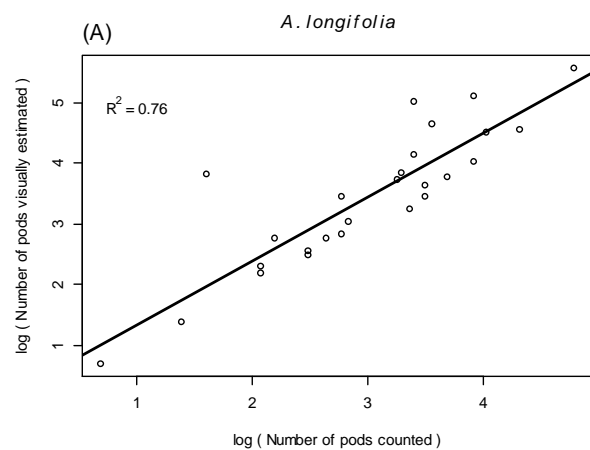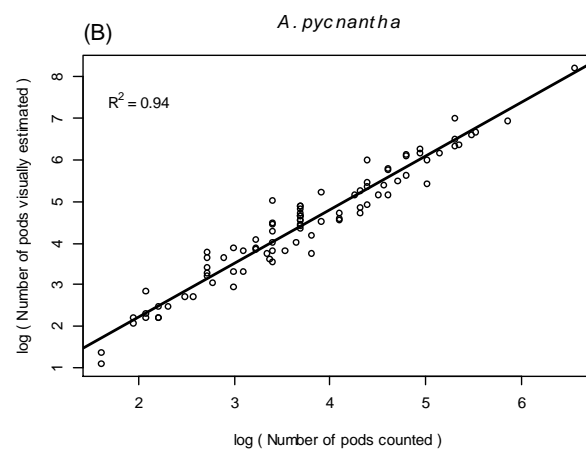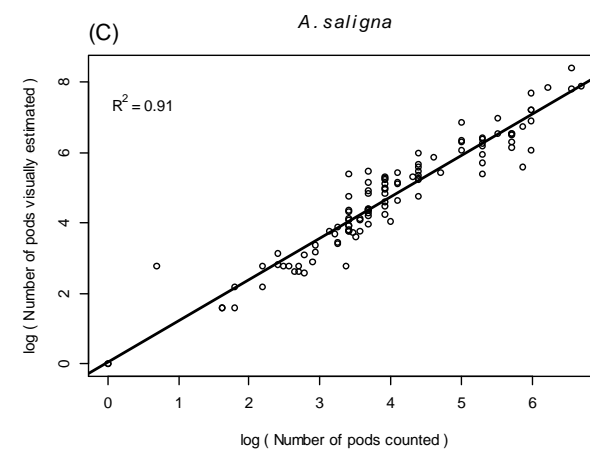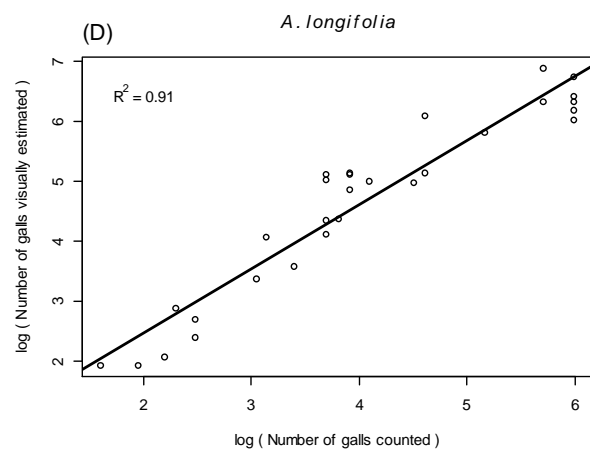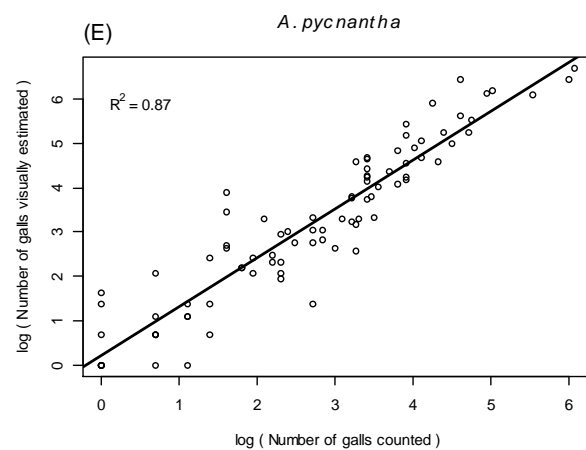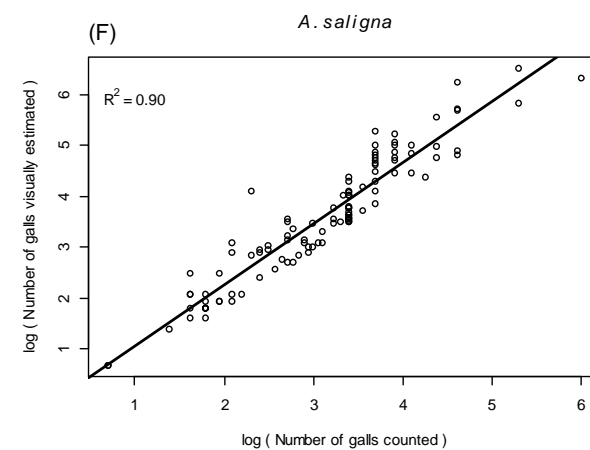

Supplement: Figure S1 — The relationship between visually estimated pods per tree and actual pods counted per tree on a log scale of three Australian Acacia in the Western Cape of South Africa are shown in graphs (A-C). The relationship between visually estimated galls per tree and actual galls counted per tree on a log scale of three Australian Acacia in the Western Cape of South Africa are shown in graphs (D-F). [file peerj-07-6816-s002.pdf]
