# Supplementary material for: Seed survival of Australian Acacia in the Western Cape of South Africa in the presence of biological control agents and given environmental variation
Source: PeerJ. 2019 Apr 29;7:e6816. doi: 10.7717/peerj.6816 (PMC6497107; doi:10.7717/peerj.6816)
Supplement: Table S1 [file peerj-07-6816-s003.docx]

| **Site** | **Species** | **Co-ordinates** | **MAP** | **WCP** |
| --- | --- | --- | --- | --- |
| Fabel | Al | S 33° 21’ E 19° 13’ | 598 | 80 |
| Mooiplaas | Al | S 33° 28’ E 19° 09’ | 598 | 80 |
| Romansrivier | Al | S 33° 28’ E 19° 12’ | 598 | 80 |
| Avondvrede | Al | S 33° 49’ E 18° 52’ | 689 | 75 |
| White River | Al | S 34° 24’ E 19° 32’ | 748 | 63 |
| Locheim | As | S 33° 13’ E 18° 39’ | 416 | 81 |
| Lio Marico | As | S 33° 45’ E 18° 46’ | 576 | 75 |
| Paarl Nantes | As | S 33° 41’ E 18° 58’ | 688 | 81 |
| Modderrivier | As | S 34° 25’ E 19° 30’ | 748 | 63 |
| Fairfield | As | S 33° 24’ E 19° 48’ | 412 | 60 |
| Vaalvlei | Ap | S 33° 28’ E 19° 12’ | 598 | 80 |
| De Liefde | Ap | S 33° 29’ E 19° 13’ | 598 | 80 |
| Rivendale | Ap | S 33° 46’ E 18° 47’ | 576 | 75 |
| Iddasvallei | Ap | S 33° 55’ E 18° 54’ | 955 | 78 |
| Squaredale | Ap | S 33° 17’ E 19° 46’ | 439 | 60 |
| Fraaigelegen | Am | S 33° 21’ E 19° 12’ | 598 | 80 |
| De Kijker | Am | S 33° 26’ E 19° 09’ | 598 | 80 |
| Rivendale | Am | S 33° 46’ E 18° 47’ | 576 | 75 |
