# Supplementary material for: Seed survival of Australian Acacia in the Western Cape of South Africa in the presence of biological control agents and given environmental variation
Source: PeerJ. 2019 Apr 29;7:e6816. doi: 10.7717/peerj.6816 (PMC6497107; doi:10.7717/peerj.6816)
Supplement: Table S2 — The proportion of the same seed crop that was lost after one year’s burial at the respective sites are also indicated. [file peerj-07-6816-s004.docx]

| **Species** | **Site** | **% Non-dormant** | **% Lost** |
| --- | --- | --- | --- |
| *A. longifolia* | Heuningbos | 27 | 78 |
| *A. mearnsii* | De Liefde | 27 | 79 |
|  | Rivendale | 22 | 74 |
| *A. pycnantha* | Vaalvlei | 11 | 49 |
|  | Rivendale | 16 | 50 |
|  | Iddasvalley | 36 | 75 |
| *A. saligna* | Locheim | 17 | 65 |
|  | Lio Marico | 16 | 65 |
|  | Modderrivier | 18 | 66 |
