# Supplementary material for: Seed survival of Australian Acacia in the Western Cape of South Africa in the presence of biological control agents and given environmental variation
Source: PeerJ. 2019 Apr 29;7:e6816. doi: 10.7717/peerj.6816 (PMC6497107; doi:10.7717/peerj.6816)
Supplement: Table S3 — Response functions were fitted through using quantile regression. [file peerj-07-6816-s005.docx]

|  | | | | |
| --- | --- | --- | --- | --- |
| **Species** | **a** | **b** | **c** | **d** |
| *A. pycnantha* | 4.52401423 | -0.03877475 | - | - |
| *A. saligna* | 3.19403580 | -0.05406187 | - | - |
| *A. longifolia* | 3.53064156 | -0.02015252 | - | - |
