# Supplementary material for: Seed survival of Australian Acacia in the Western Cape of South Africa in the presence of biological control agents and given environmental variation
Source: PeerJ. 2019 Apr 29;7:e6816. doi: 10.7717/peerj.6816 (PMC6497107; doi:10.7717/peerj.6816)
Supplement: Table S4 — Response functions were fitted through using quantile regression. [file peerj-07-6816-s006.docx]

|  | | | | |
| --- | --- | --- | --- | --- |
| **Species** | **a** | **b** | **c** | **d** |
| *A. pycnantha* | -0.75050066 | 0.01584076 | 3.46782951 | 0.17005285 |
| *A. saligna* | -1.23754518 | 0.01691589 | 3.20342147 | 0.14226313 |
| *A. longifolia* | -4.77726892 | 0.01881022 | 1.67058693 | - |
